# Supplementary material for: Identification and functional analysis of non-coding regulatory small RNA FenSr3 in Bacillus amyloliquefaciens LPB-18
Source: PeerJ. 2023 May 15;11:e15236. doi: 10.7717/peerj.15236 (PMC10194069; doi:10.7717/peerj.15236)
Supplement: Supplemental Information 4 [file peerj-11-15236-s004.zip › KO/CK-vs-T1_map/map03070.html]

KEGG PATHWAY: Bacterial secretion system - Reference pathway


|  |  |
| --- | --- |
| **Bacterial secretion system - Reference pathway** |  |

[
Pathway menu
| Organism menu
| Pathway entry
| Show description
| User data mapping
]

Gram-negative bacteria secrete a wide range of proteins whose functions include biogenesis of organelles, such as pilli and flagella, nutrient acquisition, virulence, and efflux of drugs and other toxins. Six distinct secretion systems have been shown to mediate protein export through the inner and outer membranes of Gram-negative bacteria. These pathways are highly conserved throughout the Gram-negative bacterial species. In Gram-positive bacteria, secreted proteins are commonly translocated across the single membrane by the Sec pathway or the two-arginine (Tat) pathway.

|  |  |  |
| --- | --- | --- |
| Reference pathway | 184% 150% 122% 100% 82% 67% 55% | 图片下载 |
